# The Optimization of Corrosion Performance of Al-Zn-Mg-Cu Alloy by Si Addition and Solid Solution Treatment

**DOI:** 10.3390/ma19071406

**Published:** 2026-04-01

**Authors:** Dongwei Zhang, Yi Lu, Huijun Shi, Shengping Wen, Wu Wei, Xiaolan Wu, Kunyuan Gao, Hui Huang, Xiangyuan Xiong, Peng Cao, Zuoren Nie

**Affiliations:** 1 Dongguan Xianghua Hardware Technology Co., Ltd., Dongguan 523000, China; zhangdongwei@inwood.com.cn; 2 College of Materials Science & Engineering, Beijing University of Technology, Beijing 100124, China; shihj@emails.bjut.edu.cn (H.S.); weiwu@bjut.edu.cn (W.W.); xiaolan.wu@bjut.edu.cn (X.W.); gaokunyuan@bjut.edu.cn (K.G.); huanghui@bjut.edu.cn (H.H.); xiangyuanxiong@bjut.edu.cn (X.X.); 3 Department of Chemical and Materials Engineering, The University of Auckland, Auckland 1142, New Zealand; p.cao@auckland.ac.nz

**Keywords:** GPB-II phase, overburning, potential difference, solution treatment, Si content

## Abstract

Achieving a balanced combination of mechanical performance and corrosion resistance remains a critical challenge restricting the broader application of Al–Zn–Mg–Cu alloys in aerospace, marine, and transportation industries. In this investigation, the addition of Si significantly enhances the mechanical properties of the alloy. Among them, the alloy containing 0.35Si has the best corrosion resistance, which is closely related to the transformation of precipitates. A non-monotonic relationship between Si content and corrosion resistance was observed. At low Si levels, the simultaneous precipitation of η, T, and GPB-II phases leads to a large electrochemical potential difference among these phases, which promotes micro-galvanic corrosion. With increasing Si content, the microstructure evolves toward the dominance of GPB-II precipitates, thereby reducing the internal potential difference and improving corrosion resistance. However, excessive addition of Si will lower the equilibrium solid phase temperature, resulting in overburning during the solid solution treatment process and a significant decrease in corrosion resistance. In addition, lowering the solution treatment temperature effectively improves corrosion resistance by suppressing the formation of remelted spheres and low-melting-point brittle phases along grain boundaries. These phases can form strong micro-galvanic couples with the matrix, accelerating anodic dissolution. Therefore, by adding an appropriate amount of Si and optimizing the solid solution temperature, a corrosion-resistant high-strength Al-Zn-Mg-Cu-Si alloy can be obtained. This strategy also provides a broader compositional and heat-treatment design window, which could be further expanded through the incorporation of rare-earth (RE) elements.

## 1. Introduction

Al–Zn–Mg–Cu alloys are heat treatable, with mechanical properties that can be significantly enhanced through appropriate solution treatment and aging. The strengthening effect primarily originates from the formation of finely dispersed precipitates during aging, which effectively impede dislocation motion [1]. However, the improvement in strength is frequently accompanied by a deterioration in corrosion resistance. In particular, localized corrosion phenomena, such as pitting corrosion and filiform corrosion, become more pronounced [2,3]. Consequently, achieving a balanced combination of mechanical performance and corrosion resistance remains a critical challenge restricting the broader application of Al–Zn–Mg–Cu alloys in aerospace, marine, and transportation industries [4,5].

Localized corrosion, including intergranular corrosion (IGC) and exfoliation corrosion (EXCO), is strongly affected by the alloy microstructure. IGC originates from the electrochemical potential difference between the grain boundary and the adjacent matrix. The grain boundary, typically exhibiting a lower potential, acts as the anodic site, leading to preferential dissolution and corrosion along the boundary. This process degrades alloy strength and may ultimately result in premature structural failure. EXCO is generally regarded as a severe manifestation of IGC in alloys with elongated grain structures, where corrosion products accumulate and expand along grain boundaries, causing delamination and spalling of surface layers [6].

Regulation of grain boundary precipitates and the width of the precipitate-free zone (PFZ) has been demonstrated to effectively enhance corrosion resistance [7,8]. Strategies such as creep aging treatment [9], non-isothermal aging [10], and increasing quenching rate [11] can promote the formation of discontinuous grain boundary precipitates and narrower PFZs, thereby mitigating localized corrosion. Consequently, the morphology and distribution of grain boundary-associated precipitates are critically correlated with the corrosion performance of Al–Zn–Mg–Cu alloys.

In general, two primary approaches are employed to enhance the corrosion resistance of Al–Zn–Mg–Cu alloys. One strategy involves microalloying with Ag, Cu, and rare-earth (RE) elements; the other relies on optimized heat treatment schedules, typically incorporating high-temperature over-aging and increased quenching rates. Ag additions can effectively trap vacancies and interact with Zn and Mg solute atoms, thereby promoting precipitation near grain boundaries and reducing the width of the precipitate-free zone (PFZ) [12,13,14]. Cu exhibits a similar effect, contributing to PFZ narrowing and improved corrosion resistance [8]. The incorporation of rare-earth elements, such as Er and Zr, facilitates the formation of discontinuous grain boundary precipitates, which mitigates intergranular corrosion susceptibility [15,16,17]. Nevertheless, microalloying inevitably increases material cost. Moreover, for alloys designed for specific engineering applications, modifying the type and content of microalloying elements is often impractical due to compositional constraints and industrial processing considerations.

A lower quenching rate generally results in a widened precipitate-free zone (PFZ) along grain boundaries [18,19], thereby increasing susceptibility to localized corrosion. Retrogression and re-aging (RRA) treatment, a three-step aging process, involves initial T6 aging, followed by a short-duration high-temperature retrogression stage and subsequent low-temperature re-aging [20]. This process effectively narrows the PFZ [21] and promotes a discontinuous distribution of grain boundary precipitates [22], yielding corrosion resistance comparable to that of the T76 temper state. This phenomenon is associated with the dissolution of grain boundary precipitates during the retrogression stage, allowing solute atoms to re-enter the matrix and subsequently re-precipitate near grain boundaries during re-aging [21]. Since the retrogression stage is conducted at an elevated temperature, aging precipitates tend to coarsen, which compromises the strengthening effect and leads to a reduction in alloy strength.

Si is an unavoidable impurity element in Al–Zn–Mg–Cu alloys and is difficult to eliminate during processing. Previous studies have demonstrated that Si addition can modify the precipitation sequence, accelerate precipitation kinetics [23], and consequently enhance mechanical performance [24]. In particular, Si-containing Al–Zn–Mg–Cu alloys can form a core–shell-structured GPB-II phase [25], which is characterized by fine scale, rapid formation, and improved structural stability [26], thereby contributing to enhanced strength and thermal stability. Furthermore, previous studies have reported that Si-containing alloys subjected to peak aging [17], retrogression and re-aging (RRA) [27], or two-step aging treatments (high-temperature pre-aging followed by low-temperature aging) [28] exhibit discontinuous grain boundary precipitates and significantly narrowed precipitate-free zones (PFZs), leading to improved corrosion resistance.

However, a systematic investigation of the effect of various Si content on corrosion behavior remains lacking and the influence of the phase transformation caused by Si on corrosion mechanisms are neglected. In addition, the role of solution treatment—an essential parameter in heat-treatable alloys—is often overlooked in corrosion studies. It has been reported that selecting a lower solution temperature can markedly reduce hydrogen embrittlement susceptibility, which is associated with the continuous grain boundary precipitation caused by excessively high solution temperatures [29]. Therefore, this investigation aims to elucidate the various Si content and the effect of solution treatment temperature on localized corrosion behavior, including intergranular corrosion (IGC) and exfoliation corrosion (EXCO), with the objective of achieving an optimized balance between mechanical performance and corrosion resistance. This paper also uses the change in the alloy’s precipitation sequence caused by Si to explain the reasons for the variation in the alloy’s corrosion performance.

## 2. Materials and Methods

Master alloys (Al–50 wt.% Cu and Al–20 wt.% Si), with pure Zn, pure Mg, and high-purity Al (99.99 wt.%), were used as raw materials. Alloy melting was conducted in a VBF-1200X air-circulating resistance furnace (Hefei Kejing Material Technology Compant, LTD, Hefei, China). The charge was melted at 780 ± 10 °C, held for 10 min to ensure compositional homogeneity, and then poured into a steel mold to obtain ingots with dimensions of 35 × 105 × 175 mm^3^. The actual chemical compositions were determined via X-ray fluorescence spectroscopy (XRF) (Bruker Corporation, Billerica, MA, USA), as listed in Table 1.

The as-cast ingots were homogenized at 530 °C for 10 h and subsequently hot-rolled at 470 °C into sheets with a final thickness of 3 mm, with a thickness reduction of less than 10% per pass. The rolled sheets were divided into two groups that were solution-treated at 530 °C and 540 °C for 1 h, followed by immediate water quenching. Peak aging was conducted at 150 °C, and the aged specimens were used for corrosion evaluation. Age-hardening behavior at 150 °C was monitored using a microhardness tester (HXD-1000TM/LCD, Laizhou Huayin Testing Instrument Co., Ltd., Laizhou, China). Tensile properties were measured on a universal testing machine (ETM−205D, Shenzhen Wance Testing Machine Co., Ltd., Shenzhen, China).

Intergranular corrosion (IGC) tests were performed in accordance with ASTM G110–92 [30]. The specimens were ground and polished prior to immersion in a solution containing 57 g/L NaCl and 10 mL/L H_2_O_2_ (30 wt.%), maintained at 30 ± 3 °C for 6 h. Exfoliation corrosion (EXCO) tests followed ASTM G34–01 [31]. Pre-treated specimens were immersed in a solution of 234 g/L NaCl, 50 g/L KNO_3_, and 6.3 mL/L HNO_3_ (70 wt.%) at 25 ± 3 °C for 48 h. After corrosion testing, samples were sectioned along the transverse direction, ground, and polished. The maximum corrosion depth was measured using an optical microscope (Olympus PMG3, Olympus Corporation, Tokyo, Japan). The corrosion tests for each component involve three parallel samples.

Microstructural characterization was conducted using scanning electron microscopy (SEM) and transmission electron microscopy (TEM). SEM observations of specimens subjected to different solution treatments were performed on a Gemini SEM 300 system (Carl Zeiss AG, Oberkochen, Germany). Precipitate characteristics in aged alloys were examined using a JEM-2100 TEM (JEOL Ltd., Tokyo, Japan) operated at an accelerating voltage of 200 kV. TEM foils were prepared by twin-jet electropolishing: disks with a diameter of 3 mm were punched from the sheets and subsequently thinned using a twin-jet electropolisher (MTP−1A, Shanghai Jiao Tong University, Shanghai, China).

## 3. Results

### 3.1. Tensile Properties and Age-Hardening Behavior

**Figure 1:**
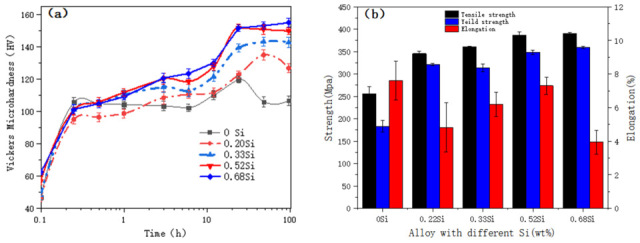
Age-hardening curve and tensile properties of the alloy at 150 °C. (**a**) Isothermal aging; (**b**) tensile properties in the peak aging state.

Figure 1 presents the age-hardening curves at 150 °C and the corresponding tensile properties at peak-aged condition. As shown in Figure 1a, the addition of Si markedly enhances the peak hardness. The alloy containing 0.5 wt.% Si exhibits the highest hardness, indicating that the solid-solution effect of Si approaches saturation at this composition. The peak hardness increases from 119.9 HV for the Si-free alloy to 152 HV with 0.5 wt.% Si, corresponding to an increment of approximately 32 HV. On average, each 0.1 wt.% increase in Si content results in a hardness increment of about 6.4 HV.

The tensile properties, shown in Figure 1b, display a strength evolution consistent with the hardness variation. However, excessive Si addition (0.7 wt.%) leads to a noticeable reduction in elongation, suggesting a trade-off between strength enhancement and ductility at higher Si levels.

### 3.2. Corrosion Behavior

#### 3.2.1. Effect of Solution Temperature on Exfoliation Corrosion Behavior

**Figure 2:**
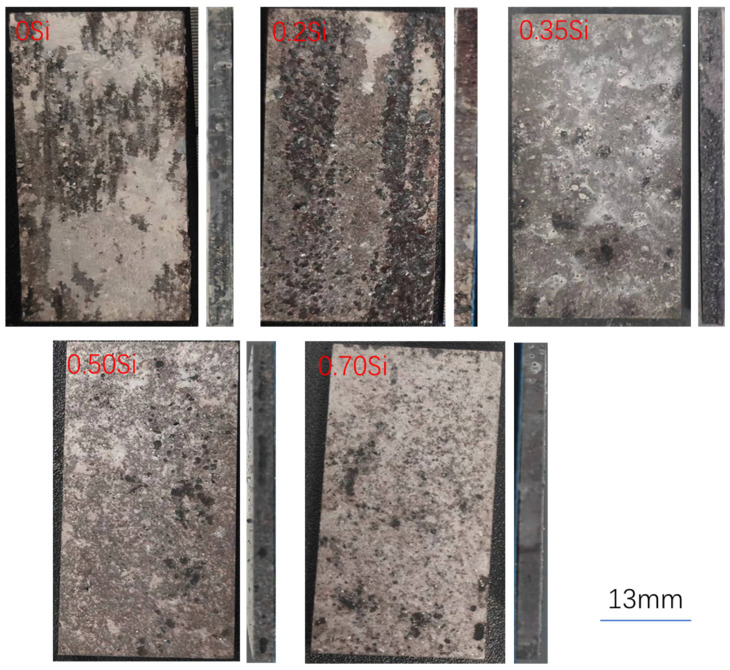
Surfaces of five alloys after exfoliation corrosion under solution treatment at 530 °C and peak aging at 150 °C.

Figure 2 shows the surface morphologies of peak-aged alloys after exfoliation corrosion (EXCO) testing following solution treatment at 530 °C. The Si-free alloy exhibits a non-uniform exfoliated layer, indicating heterogeneous corrosion susceptibility among different grains. In the alloy containing 0.2 wt.% Si, severe surface exfoliation is observed, with variations in exfoliation depth (reflected by color contrast), suggesting microstructural and compositional inhomogeneity. These results indicate that minor Si addition at this level deteriorates exfoliation corrosion resistance. In contrast, alloys with Si contents above 0.35 wt.% display markedly suppressed exfoliation, characterized primarily by sparse, uniformly distributed localized attack rather than extensive surface delamination. Cross-sectional observations reveal no pronounced lamellar separation in any of the alloys. Therefore, under a solution treatment temperature of 530 °C, low Si addition is detrimental to exfoliation corrosion resistance, whereas increasing the Si content beyond 0.35 wt.% effectively mitigates exfoliation damage.

**Figure 3:**
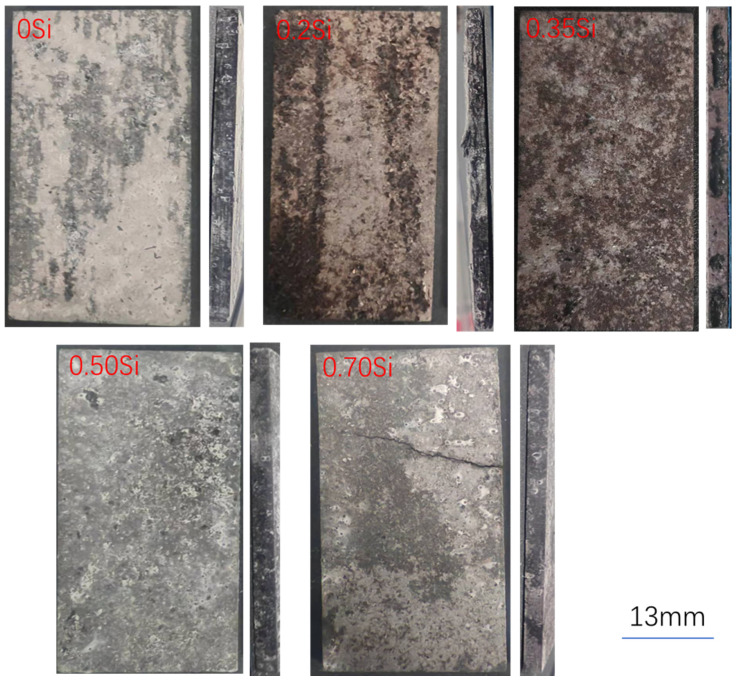
Surfaces of five alloys after exfoliation corrosion under solution treatment at 540 °C and peak aging at 150 °C.

Figure 3 presents the surface morphologies of peak-aged alloys subjected to exfoliation corrosion (EXCO) testing after solution treatment at 540 °C. In comparison with Figure 2, the regulation of the five alloys’ surface trends is broadly similar to those observed for alloys solution-treated at 530 °C followed by peak aging. However, pronounced differences are evident in the cross-sectional morphology. The alloy containing 0.2 wt.% Si exhibits severe delamination and layer separation, with the exfoliated layer showing noticeable expansion, and exfoliation damage also extends into the interior of the rolled sheet. Although the 0.7 wt.% Si alloy does not display obvious lamellar separation, long surface cracks are observed. The other three alloys also show a decline in exfoliation corrosion resistance compared with those solution-treated at 530 °C, indicating that the higher solution temperature adversely affects corrosion performance to varying degrees.

#### 3.2.2. Effect of Solution Treatment on Intergranular Corrosion Behavior

**Figure 4:**
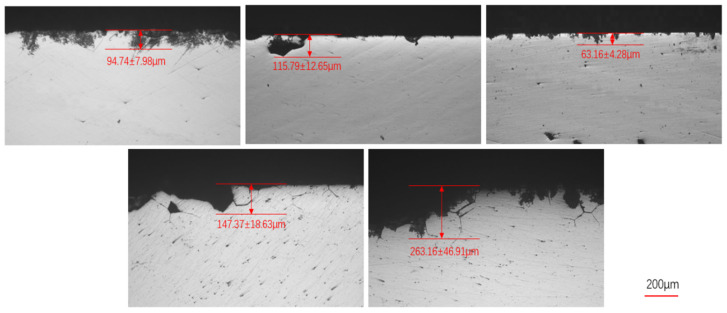
Intergranular corrosion depth of five alloys under solution treatment at 530 °C and peak aging at 150 °C.

Figure 4 summarizes the intergranular corrosion (IGC) depths of the five alloys after solution treatment at 530 °C followed by peak aging. The results indicate that the addition of 0.35 wt.% Si leads to the minimum IGC depth, demonstrating improved resistance to intergranular corrosion. In contrast, both insufficient and excessive Si additions increase the IGC depth. When the Si content exceeds 0.5 wt.%, the grain boundary corrosion resistance deteriorates noticeably, with the 0.7 wt.% Si alloy exhibiting the most severe intergranular attack.

**Figure 5:**
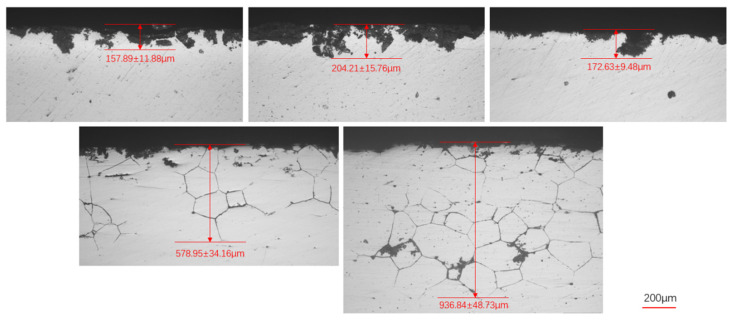
Intergranular corrosion depth of five alloys under solution treatment at 540 °C and peak aging at 150 °C.

Figure 5 presents the intergranular corrosion (IGC) depths of the five alloys after solution treatment at 540 °C followed by peak aging. Similar to the trend observed in Figure 4, the alloy containing 0.35 wt.% Si exhibits the lowest IGC depth, indicating superior resistance to intergranular attack. However, when the Si content exceeds 0.5 wt.%, the IGC depth increases markedly, accompanied by grain boundary corrosion features.

Overall, the solution treatment temperature plays a critical role in governing corrosion behavior. Higher solution temperature adversely affects both intergranular corrosion and exfoliation corrosion resistance. Therefore, optimization of the solution treatment temperature is essential for achieving improved corrosion performance in practical applications.

### 3.3. Microstructural Characterization

#### 3.3.1. SEM Observations After Various Solution Treatments

According to the corrosion results, Alloy 2 exhibits inferior exfoliation corrosion resistance compared with the other alloys, irrespective of whether solution treatment was conducted at 530 °C or 540 °C. In addition, Alloys 4 and 5 show a pronounced deterioration in intergranular corrosion resistance after solution treatment at 540 °C. To elucidate the origin of these differences, SEM and TEM analyses were performed on Alloys 2, 4, and 5, which were subjected to different solution treatment temperatures.

**Figure 6:**
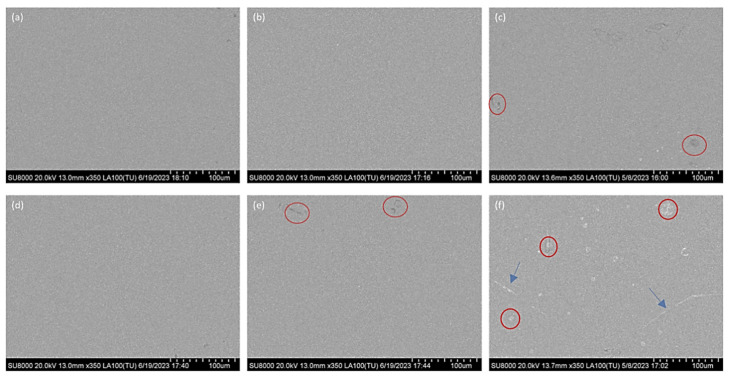
SEM images of the alloy after different solid solution treatment temperatures. (**a**,**d**) Alloy 2; (**b**,**e**) Alloy 4; (**c**,**f**) Alloy 5 ((**a**–**c**) 530 °C, (**d**–**f**) 540 °C).

Figure 6 presents the SEM micrographs of the three alloys after solution treatment at 530 °C and 540 °C. For Alloy 2, primary phases are completely dissolved at both temperatures, and no overburning features are observed. In contrast, Alloy 4 exhibits dark remelting droplets (red circle) at 540 °C, indicative of overburning. Alloy 5 shows remelting particles even at 530 °C, while more severe grain boundary overburning (blue arrow) accompanied by remelting droplets (red circle) is evident at 540 °C. These observations demonstrate that overburning occurs in Alloys 4 and 5 at 540 °C, which is closely associated with the degradation in corrosion resistance. The results further suggest that Si content influences the overburning threshold temperature, thereby indirectly affecting the corrosion behavior of the alloys.

**Figure 7:**
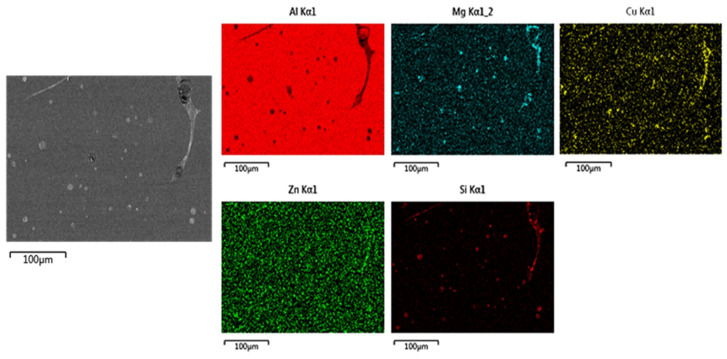
SEM elemental mapping of Alloy 5 after solution treatment at 540 °C.

Elemental mapping analyses were performed on the grain boundary phases and remelting droplets, as shown in Figure 7. The grain boundary regions are predominantly enriched in Zn, Mg, Cu, and Si, whereas the remelting droplets are mainly enriched in Mg, Si, and Cu. Combined with the observations in Figure 6e,f, it is evident that increasing Si content promotes the formation of grain boundary phases. The intragranular remelting droplets are primarily identified as Mg_2_Si-type phases, which are commonly observed in 6xxx series alloys. The melting point of Mg_2_Si-type phases is higher than that of the η or T phases. Owing to their relatively low melting point, these phases are prone to melting and overburning at elevated solution treatment temperatures.

#### 3.3.2. Precipitate Characteristics in Peak-Aged Alloys

The results shown in Figure 2, Figure 3, Figure 4 and Figure 5 indicate that as the Si content increases, the corrosion performance initially decreases, then increases, and finally decreases again. Previous studies have shown that the addition of Si can reduce the width of the PFZ, but it has little effect on the continuity of the grain boundary precipitates [17,27,28,29]. This theory cannot explain the fluctuating changes in the corrosion performance of the Si-containing alloys observed in this investigation. Therefore, we expected to explain the cause of the fluctuation in corrosion performance through the variation (potential) of the alloy’s precipitates.

**Figure 8:**
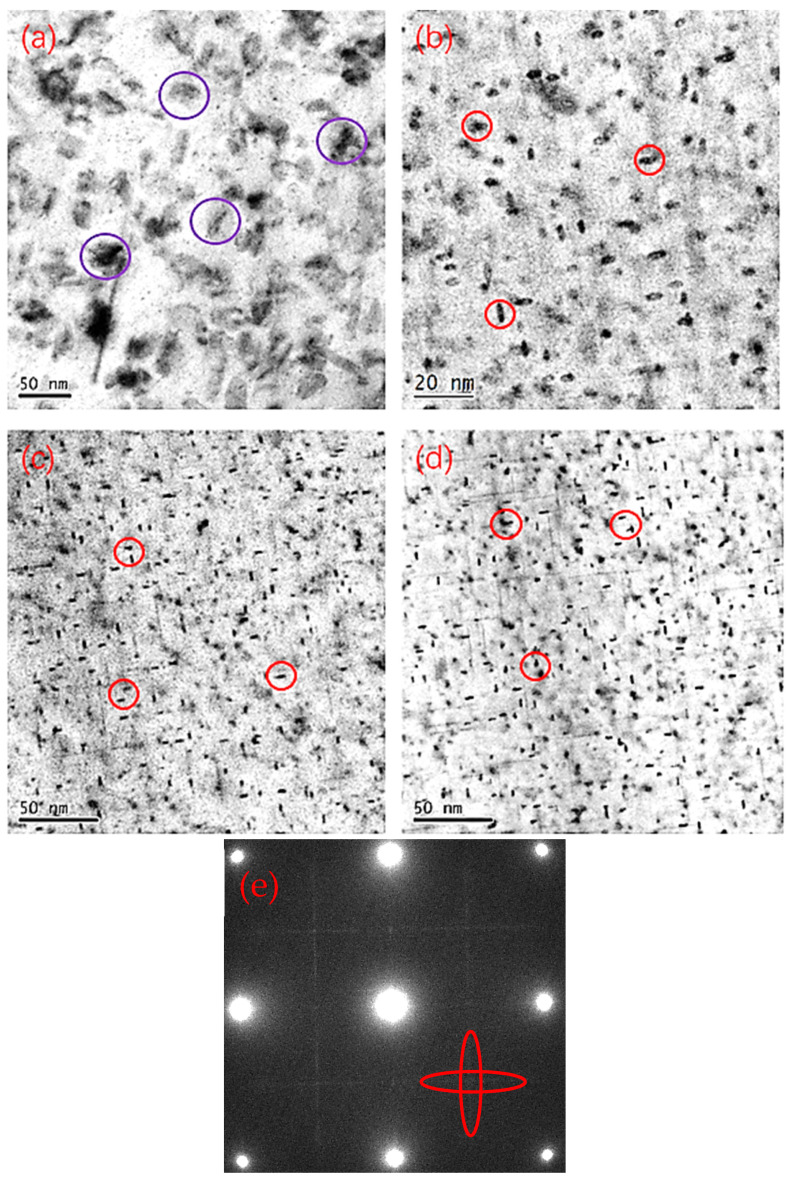
TEM and corresponding SAED images of peak aged state alloys. (**a**,**b**) Alloy 2; (**c**) Alloy 3; (**d**) Alloy 4; (**e**) SAED pattern of Alloy 4.

Figure 8 presents the TEM micrographs of the three alloys at the peak-aged condition. The diffraction patterns of the three alloys are similar, so we choose Alloy 4 as the representative. As can be seen from Figure 8e, the cross-shaped diffraction pattern is observed, which indicates that the alloy contains the GPB-II phase. In Alloy 2, a large number of η and T phases (purple circle) are observed, with an average size of 13 nm. At higher magnification, fine GPB-II precipitates (red circle) can also be identified within the matrix. In contrast, Alloys 3 and 4 predominantly contain GPB-II precipitates (red circle), whose dimensions are significantly smaller than those of the η and T phases. These results indicate that Si content markedly influences the precipitation sequence and phase transformation. The evolution of precipitate type and size distribution is therefore responsible for the observed variations in mechanical properties.

## 4. Discussion

### 4.1. Effect of Si Content on Precipitation Behavior and Mechanical Properties

The incorporation of Si alters the precipitation sequence and phase types of the alloy. With increasing Si content, the number density of η and T phases decreases, whereas that of GPB-II precipitates increases.

Si exhibits a strong affinity for Mg, Cu, and vacancies, facilitating the formation of Mg–Si–(Cu)–vacancy clusters. These clusters are energetically more favorable than Mg–Zn or Mg–Zn–Cu clusters and therefore form preferentially during the early stage of aging [23]. With prolonged aging, these clusters evolve into L-phase cores, which subsequently act as heterogeneous nucleation sites for GPB zones [26]. This process ultimately leads to the formation of core–shell-structured GPB-II precipitates, consistent with previous reports [25].

Compared with η and T phases, GPB-II precipitates provide superior age-hardening efficiency due to their finer scale and higher number density, thereby significantly enhancing mechanical strength. However, excessive Si addition results in the formation of continuous brittle phases along grain boundaries after solution treatment, which deteriorates ductility and reduces elongation.

### 4.2. Effect of Si Content on Solution Treatment Temperature

**Figure 9:**
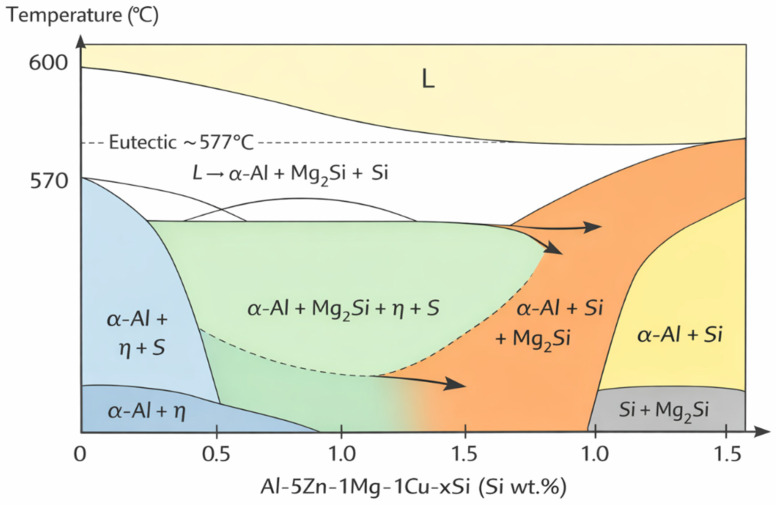
Quasi-binary cross-section schematic diagram of the Al-5Zn-1Mg-1Cu-xSi (wt.%) system.

In the Si-containing Al–5Zn–1Mg–1Cu alloy system, increasing solute content promotes the formation of low-melting eutectic phases and intermetallic compounds, leading to a reduction in the equilibrium solidus temperature as shown in Figure 9. The addition of Si further expands the Mg-Si phase field and aggravates interdendritic segregation, thereby decreasing the remelting temperature. Moreover, the thermodynamic contraction of the α-Al solid solution region further lowers the permissible solution treatment temperature. Furthermore, an increase in the solid solution temperature will enhance the diffusion rate of the solute element. Therefore, when the alloy is solid-solved at a higher temperature, the segregation is likely to occur at the grain boundaries, thereby forming grain boundary overburned phases.

Table 2 summarizes the temperatures corresponding to the endothermic peaks in the DSC curves for alloys with different Si contents as shown in Figure S1. The temperature corresponding to the DSC endothermic peak indicates that the primary phase within the alloy begins to dissolve, and the overburning temperature of the alloy should be lower than this temperature. As the Si content increases, the characteristic peak temperature shifts to lower values. This peak temperature corresponds to the melting of primary or eutectic phases in the as-cast microstructure. The downward shift therefore confirms that Si addition promotes the formation of low-melting eutectic and intermetallic phases, ultimately reducing the allowable solution treatment temperature.

### 4.3. Effects of Si Content and Solution Temperature on Corrosion Behavior

Table 3 summarizes the corrosion and mechanical properties of the Al-Zn-Mg-Cu-(Si) alloy [17,27]. The strength of the Al-5Zn-1Mg-1Cu-Si alloy is higher than that of the Al-4.5Zn-1.5Mg-1Cu-Si alloy. This experimental alloy has a more excellent EXEO grade, and its IGC is comparable to that of previous studies. Future research can further enhance the corrosion performance of the alloy by adding Er.

The addition of a minor amount of Si initially deteriorates both exfoliation corrosion (EXCO) and intergranular corrosion (IGC) resistance. This degradation is closely associated with the concurrent precipitation of η, T, and GPB-II phases. Previous studies have found that adding Si to alloys can increase the self-corrosion potential and reduce the self-corrosion current density. This is because the corrosion potential of GPB-II phase is higher than that of the η and T phases [27]. The potential from high to low is GPB-II phase > T phase > η phase. These precipitates exhibit distinct electrochemical potentials; notably, η and T phases possess lower potentials than GPB-II, resulting in a larger potential difference and a stronger tendency to act as anodic sites, thereby undergoing preferential dissolution.

In the Si-free alloy, only η and T phases are present. Although their number density is higher, the electrochemical potential difference between these two phases is relatively small, leading to a lower driving force for anodic dissolution and consequently a reduced corrosion rate compared with alloys containing a small amount of Si.

With further increase in Si content, the precipitates gradually transform from η and T phases to GPB-II precipitates. Owing to their relatively higher electrochemical potential and the reduced potential heterogeneity in a microstructure dominated by GPB-II, galvanic interactions are mitigated, resulting in improved corrosion resistance.

Overall, in alloys with a lower Si content, the η phase acts as the anode. Due to the presence of the GPB-II phase, the potential difference in the alloy increases, which leads to a decline in the alloy’s corrosion performance. In the 0.35Si alloy, there is only the GPB-II phase. There is almost no potential difference in this alloy and the overall potential of the alloy is relatively high. So the corrosion resistance of 0.35Si alloy has been enhanced.

However, excessive Si addition induces overburning during solution treatment, giving rise to remelting droplets and microstructural inhomogeneities. These solute-enriched spherical particles and the adjacent solute-depleted regions generate pronounced micro-galvanic coupling, which accelerates localized anodic dissolution and significantly degrades corrosion resistance.

Increasing the solution treatment temperature further enlarges the volume fraction of remelting droplets and may even lead to the formation of brittle phases distributed along grain boundaries. Overburning causes incipient melting and resolidification at grain boundaries, forming continuous low-melting intermetallic compounds. The substantial electrochemical potential difference between these phases and the α-Al matrix promotes micro-galvanic activity. Consequently, the combined effect of a continuous intergranular network and enhanced electrochemical heterogeneity markedly reduces the overall corrosion resistance of the alloy.

In conclusion, alloys with higher strength and moderate corrosion property by controlling the Si content was obtained. In the future, rare-earth elements (Zr, Er) can be added to the alloys to further improve their corrosion resistance, thereby expanding the heat treatment and composition windows of the alloys.

## 5. Conclusions

Transmission electron microscopy (TEM) and scanning electron microscopy (SEM) were employed to investigate the influence of Si content and solution treatment temperature on the corrosion behavior of Si-containing Al–Zn–Mg–Cu alloys. The main findings are summarized as follows:

1. Si addition significantly enhances mechanical properties; 0.35 wt.% Si can improve corrosion resistance.
2. The corrosion response exhibits a non-monotonic dependence on Si content: a minor Si addition deteriorates exfoliation corrosion (EXCO) and intergranular corrosion (IGC) resistance, whereas an appropriate increase in Si to 0.35 wt.% improves corrosion resistance; excessive Si, however, markedly degrades it.
3. At low Si levels, η, T, and GPB-II precipitates coexist, generating electrochemical heterogeneity. The η phase, T phase possessing lower potential, preferentially acts as the anodic site and accelerates dissolution. With increasing Si content, GPB-II becomes dominant, reducing the potential difference with the α-Al matrix and thereby mitigating galvanic interactions. Excessive Si induces overburning during solution treatment, and the resulting microstructural inhomogeneity impairs corrosion resistance.
4. Elevated solution treatment temperature further deteriorates EXCO and IGC resistance due to overburning. Remelting droplets and low-melting grain boundary phases intensify micro-galvanic coupling, accelerate anodic dissolution, and facilitate corrosion propagation.

## Figures and Tables

**Table 1:** The chemical compositions of experimental alloys (wt.%).

Samples	Verified Composition (wt.%)				
	Zn	Mg	Cu	Si	Al
1# (Al-5Zn-1Mg-1Cu)	4.75	1.06	0.976	-	Bal.
2# (Al-5Zn-1Mg-1Cu-0.2Si)	4.83	1.03	1.06	0.20	Bal.
3# (Al-5Zn-1Mg-1Cu-0.35Si)	4.85	0.985	0.928	0.33	Bal.
4# (Al-5Zn-1Mg-1Cu-0.5Si)	4.84	1.03	0.98	0.52	Bal.
5# (Al-5Zn-1Mg-1Cu-0.7Si)	4.88	0.997	0.93	0.68	Bal.

**Table 2:** DSC endothermic peak temperatures for various Si content alloys.

Si Content (wt.%)	Temperature (K)
0	860.5
0.35	813.9
0.5	804.8

**Table 3:** Summary of corrosion and mechanical properties of Al-Zn-Mg-Cu-(Si) alloys.

Alloy (Al/Zn/Mg/Cu/Si/Er)	Yield Strength (MPa)	IGC Depth (μm)	Ecorr (V)	Icorr (μA/cm^2^)	EXCO Level	Ref.
4.5/1.5/1/0/0-T6	301	264.2	−0.80 ± 0.02	2.97 ± 0.11	EB	[17]
4.5/1.5/1/0.35/0.1-T6	339.5	9.9	−0.83 ± 0.02	1.52 ± 0.07	EA	[17]
4.5/1.5/1/0/0-T6	382	93	−1.28	1.82	EB	[27]
4.5/1.5/1/0.35/0-T6	352	41	−1.13	0.22	EB	[27]
4.5/1.5/1/0/0-RRA	333	12	−1.27	1.00	EA	[27]
4.5/1.5/1/0.35/0-RRA	417	20	−0.97	0.18	EA	[27]
5/1/1/0/0-T6 (530 °C)	183.18	94.74	-	-	EB	-
5/1/1/0.2/0-T6	321.41	115.79	-	-	EC	-
5/1/1/0.35/0-T6	313.2	63.16	-	-	EA	-
5/1/1/0.5/0-T6	348.29	147.37	-	-	EA	-
5/1/1/0.7/0-T6	359.25	263.16	-	-	EB	-

## Data Availability

The original contributions presented in this study are included in the article/Supplementary Materials. Further inquiries can be directed to the corresponding authors.

## Supplementary Materials

The following supporting information can be downloaded at: https://www.mdpi.com/article/10.3390/ma19071406/s1, Figure S1: DSC curve of various alloys.
