# Supplementary material for: The Optimization of Corrosion Performance of Al-Zn-Mg-Cu Alloy by Si Addition and Solid Solution Treatment
Source: Materials (Basel). 2026 Apr 1;19(7):1406. doi: 10.3390/ma19071406 (PMC13075086; doi:10.3390/ma19071406)
Supplement: Supplementary file 1 [file materials-19-01406-s001.zip › materials-4199774-supplementary.pdf]

Supplementary File

The optimization of corrosion performance of Al-Zn-Mg-Cu alloy by Si addition and solid solution treatment

Dongwei Zhang [a], Yi Lu [b, *], Huijun Shi [b], Shengping Wen [b, *], Wu Wei [b], Xiaolan Wu [b], Kunyuan Gao [b], Hui Huang [b], Xiangyuan Xiong [b], Peng Cao [c], Zuoren Nie [b, *]

[a] Dongguan Xianghua Hardware Technology Co., LTD

[b] College of Materials Science & Engineering, Beijing University of Technology, Beijing, 100124, China

[c] Department of Chemical and Materials Engineering, The University of Auckland, Auckland, 1142, New Zealand

* Corresponding author.

Yi Lu：

E-mail address: bbluyi@yeah.net

Shengping Wen:

E-mail address: wensp@bjut.edu.cn

Tel: +86 13552521441

Zuoren Nie:

E-mail address: zrnie@bjut.edu.cn

Alloy 1

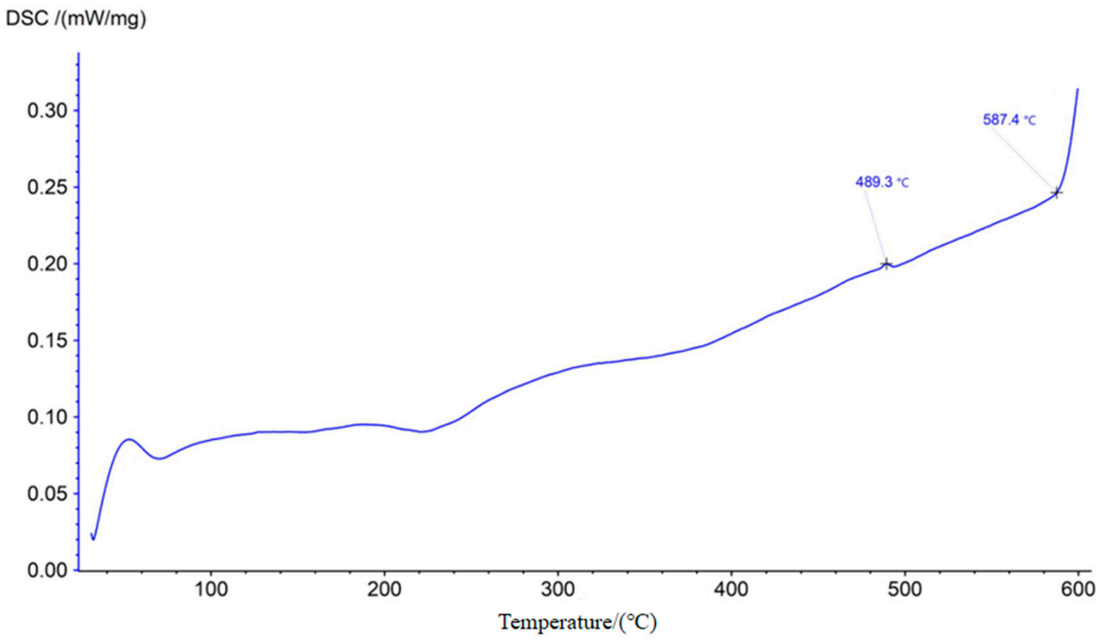

Alloy 3

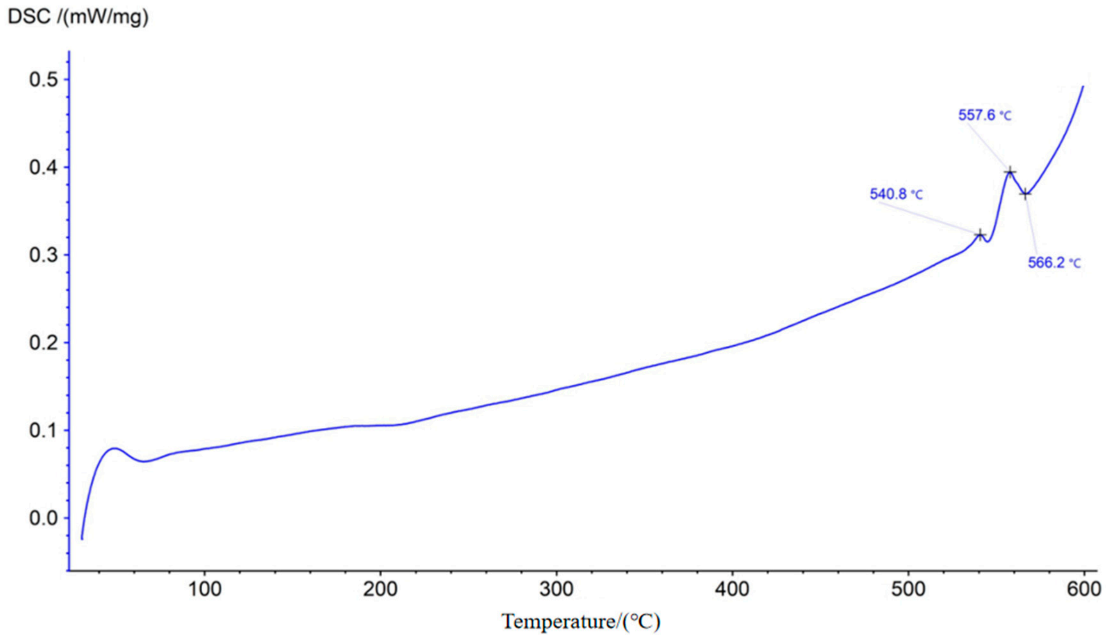

Alloy 4

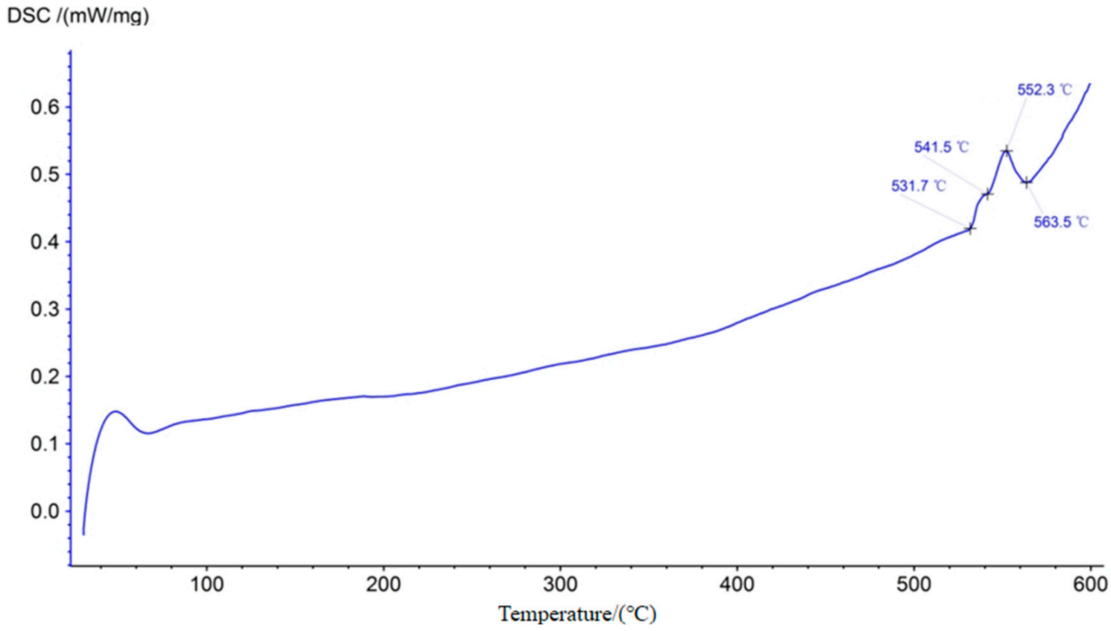

Figure S1. DSC curve of various alloys.
